# Oxidative coupling of bio-alcohols mixture over hierarchically porous perovskite catalysts for sustainable acrolein production

**DOI:** 10.1039/d1ra05627a

**Published:** 2021-08-31

**Authors:** Al-Shaimaa M. Essehaity, Dalia R. Abd ElHafiz, Delvin Aman, Sara Mikhail, Yasser K. Abdel-Monem

**Affiliations:** Catalysis Laboratory, Refining Department, Egyptian Petroleum Research Institute (EPRI) Nasr City 11727 Cairo Egypt delvin.aman@epri.sci; EPRI-Nanotechnology Center, Egyptian Petroleum Research Institute (EPRI) Nasr City 11727 Cairo Egypt; Department of Chemistry, Faculty of Science, Menoufia University 32511 Shebin El-Kom Menoufia Egypt

## Abstract

The acrolein production from bio-alcohols methanol and ethanol mixtures using AMnO_3_ (since A = Ba and/or Sr) perovskite catalysts was studied. All the prepared samples were characterized by XRD, XPS, N_2_ sorption, FTIR, Raman spectroscopy, TEM, SEM, TGA, and NH_3_–CO_2_-TPD. The catalytic oxidation reaction to produce acrolein has occurred *via* two steps, the alcohols are firstly oxidized to corresponding aldehydes, and then the aldol is coupled with the produced aldehydes. The prepared perovskite samples were modified by doping A (Sr) position with (Ba) to improve the aldol condensation. The most catalytic performance was achieved using the BaSrMnO_3_ sample in which the acrolein selectivity reached 62% (*T* = 300 °C, MetOH/EtOH = 1, LHSV = 10 h^−1^). The increase in acrolein production may be related to the high tendency of BaSrMnO_3_ toward C–C coupling formation. The C–C tendency attributes to that modification have occurred in acid/base sites because of metal substitution.

## Abstract 2

The figure illustrates the potential of perovskite as a catalyst for acrolein production *via* simultaneous oxidative coupling of renewable alcohols (ethanol and methanol) followed by aldol condensation of pre-formed aldehydes to form acrolein.

## Graphical abstract

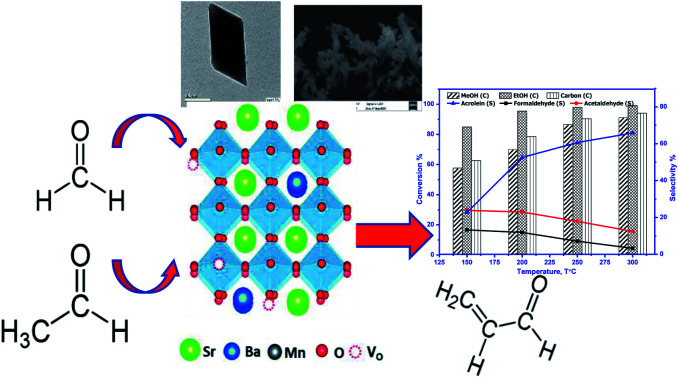

## 1. Introduction

Acrolein (CH_2_=CHCHO) is a significant chemical intermediate with high reactivity due to a combination of vinyl and carbonyl groups.^1^ Acrolein is used in many syntheses for high-value industrial products, including acrylic resins, super absorbing polymers, detergents, as well as for feed applications such as methionine and biocides, and so forth.^2^ The first commercial method for acrolein production was an aldol condensation of acetaldehyde and formaldehyde. In the 1940s, Shell developed this method using copper oxide loaded on silicon carbide as a catalyst. The industrial synthesis of acrolein is primarily carried out through propylene oxidation over bismuth, iron, molybdate, Ni and/or Co and K, including many additives Sb, B, W, or P as multi-metals mixture catalysts to improve the activity and selectivity of the prepared catalysts. Using these catalysts to achieve *ca.* 99% propylene conversion and high selectivity toward the desired product, acrolein reaches up to 80–90%.^3^ So an efficient process for the production of acrolein from renewable resources is most necessary. Accordingly, an alternative to acrolein production using a methanol/ethanol mixture as a feedstock reactant has been investigated *via* oxidative alcoholic coupling.^4^ These feedstocks can be obtained from renewable resources at low prices. Ethanol is already being fermented using three primary sources: agriculture, forestry, and industrial byproducts, while methanol from waste gasification *via* syngas conversion.^5^

In the oxidative alcoholic coupling, the reaction can be carried out in two steps as defined (eqn (1) and (2)), including partially alcohol oxidation followed by aldol condensation to acrolein.^1,6^ The reactions preferably occur in one reactor (allowing energy saving) in which all reactions occur simultaneously.

1 CH_3_OH + C_2_H_5_OH + O_2_ → HCHO + CH_3_CHO + 2H_2_O

2 HCHO + CH_3_CHO → CH_2_=CHCHO + H_2_O

Numerous catalysts have been reviewed for the gas-phase aldolization of acetaldehyde and formaldehyde in an O_2_-free environment: mixed oxides,^4^ hydrotalcite,^7^ zeolites,^8^ clays,^9^ silica, and alumina.^10^ The aldolization reaction is favored by cooperation between the acidic and basic sites (amphoteric catalysts). Therefore, it is essential that balance acidic and basic properties. Lilić *et al.*^1^ deliberated the influence of acid/base properties of numerous catalysts on the cross-aldolization of acetaldehyde and formaldehyde from a mixture of methanol and ethanol. The authors proposed that the coexistence of basic and acidic sites appears to be the optimal surface configuration for maximizing acrolein production.

Perovskites are complex mixed metallic oxides with the general formula of ABO_3,_ which have exceptional diversity of physical and chemical properties. These perovskite compounds are ceramic materials with a hexagonal structure and have a space group of *P*6_3_/*mmc* with a shared oxygen polyhedron at room temperature. Their structures can expose to tilting of the oxygen octahedrons, which causes a slight distortion of cubic perovskite structures.^11^ The defects or even spatial distortion in perovskite structure will be established when A and B elements are doped or substituted with others to form the flexible various structure oxides with the formula of 
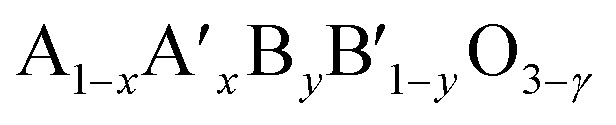
 (ref. 12) with often oxygen vacancy (*γ*). The oxygen vacancy formed and improved electronic conductivity because the doping in the A site, while the doping occurs at the B site, can promote catalytic activity.^13^ However, a great potential proposed that the oxygen vacancies in the perovskite oxide structures play a prominent role in catalytic reactions since oxygen vacancies are preferential sites for O_2_ adsorption.

From this point of view, perovskites showed significant air pollution abatement *via* the pollutant catalytic oxidation reaction. Recently, a hexagonal structure SrMnO_3_ and SrMnBO_3_ (B–Co, Cu) nano perovskite as a catalyst exhibiting high catalytic activity towards soot oxidation, which explained from XPS results (O 1s peak), SrMnCoO_3_, the highest active catalyst up to ∼ 64% because of having the highest mobility of adsorbed oxygen species.^14^

To the best of our knowledge, the only two publications exhibit applications of perovskite as a catalyst for aldol reactions were reported by Torres-Martínez *et al.*,^15^ who using alkali tantalates (ATaO_3_, with A = Li, Na, and K) for catalytic aldol condensation of acetone at the gas phase by, and Kleineberg *et al.*^16^ who exhibit the material series A = Ca, Sr and Ba, and B = Ti, Zr, and Ce as a catalyst towards aldol addition of isobutyraldehyde to formaldehyde.

Herein, we present the potential of perovskite as a catalyst towards acrolein production through the simultaneously oxidative coupling of renewable alcohols (ethanol & methanol) followed by aldol condensation of pre-formed aldehydes. The examined perovskites are AMnO_3,_ where A = Ba or/and Sr are prepared hydrothermally. The prepared perovskite samples were physically and chemically characterized. Their catalytic activity was tested in a fixed bed reactor in the gas phase.

## 2. Experimental

### 2.1. Materials used

The materials used are manganese(ii) nitrate tetrahydrate (Mn(NO_3_)_2_·4H_2_O, 99.99%, Sigma Aldrich), barium(ii) chloride dihydrate (BaCl_2_·2H_2_O, 99%, Loba Chemie), potassium permanganate (KMnO_4_, 99%, Sigma Aldrich), strontium(ii) nitrate (Sr(NO_3_)_2_, 99.99%, Sigma Aldrich) and potassium hydroxide (KOH, 85%, Loba Chemie).

### 2.2. Catalyst preparation

The BaMnO_3_ (BMO), SrMnO_3_ (SMO), and BaSrMnO_3_ (BSMO) nanoparticles perovskites were prepared by hydrothermal synthesis. Mn(NO_3_)_2_·4H_2_O and BaCl_2_·2H_2_O and Sr(NO_3_)_2_ were used as precursor materials; KMnO_4_ was used as an oxidizer, while KOH served as a mineralizer. Those precursor materials were dissolved in deionized water, and KOH was added while stirring to adjust the alkalinity of the solution.

The initial molar ratios of the input species were 0.05 KMnO_4_: 0.05 Mn(NO_3_)_2_: 0.1 (BaCl_2_ or Sr(NO_3_)_2_): 0.035 KOH for preparing BaMnO_3_ (BMO) or SrMnO_3_(SMO). While for Ba_0.5_Sr_0.5_MnO_3_ (BSMO), the initial molar ratios of the input species were 0.05 KMnO_4_: 0.05 Mn(NO_3_)_2_: (0.05 BaCl_2_ and 0.05 Sr(NO_3_)_2_): 0.035 KOH. The preparation reaction was done mainly in a Teflon vessel. The Teflon vessel was filled to 80% of its volume, and then this vessel was placed in the stainless-steel container (autoclave). The crystallization reaction occurred at 270 °C for 30 hours; after the preparation, the autoclave was cooled in air and depressurized. The solid products were washed with deionized water and dried in an oven at 100 °C overnight. A nano perovskite powder was finally obtained.

### 2.3. Catalyst characterization

The crystallographic structure and chemical composition of the prepared perovskites were investigated using X-ray powder diffraction (XRD) carried out on Shimadzu XD-1 diffractometer in 2*θ* range between 20 and 80° with Cu Kα radiation (*λ* = 1.54056 Å). By employing the well-known Scherrer relation (eqn (3)), the average crystallite size of perovskite samples can be calculated.3
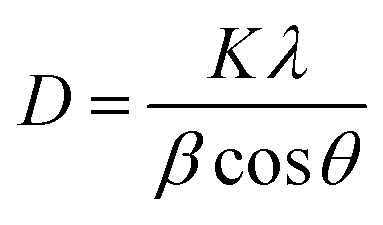
*D* is the crystal diameter, *k* is constant, *λ* is the wavelength of X-rays used for diffraction, *θ* is Bragg's angle in degrees, and *β* is the full width at half maxima peak height in radians.

The morphology of the samples was characterized by high-resolution transmission electron microscopy (HR-TEM) spectroscopy. The TEM images were obtained on a JEOL 2100F operating at 200 kV. The prepared samples were suspended in ethanol for about 30 min under ultrasonic treatment, then depositing on carbon-film-coated copper grids. N_2_ adsorption–desorption isotherms and pore size distribution were derived from the low-temperature nitrogen (−196 °C) on a gas sorption analyzer Quantachrome NOVA2000, USA. The samples were evacuated at 350 °C for 24 h before nitrogen adsorption. The coke content of the used catalysts was determined using thermal gravimetric analysis (TGA) on SDTQ-600 (TA-USA) Thermo Balance instrument. The infrared absorption spectra are presented (at 25 °C) for characterizing the functional groups in the frequency range from 400–4000 cm^−1^ with a resolution of 4 cm^−1^ on the (Bruker Tensor-27, Germany). SEM analysis was performed on Carl Zeiss (Germany) to understand the surface morphological properties of the samples, and also, atomic elemental compositions (%) were estimated from EDS analysis. X-ray photoelectron spectroscopy (XPS) analysis was undertaken by Al-Kα micro-focused monochromator XPS spectrometer.

The structure of the prepared perovskite samples can be tuned in numerous substitutes by other cations. However, perovskites stability is determined by the Goldschmidt tolerance factor^17^ (eqn (4)).4
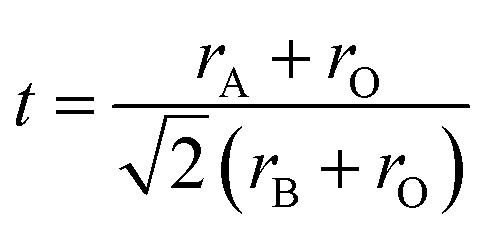
*r*_A_, *r*_B_ and *r*_O_ represent the ionic radii of A and B cations and O anion of ABO_3_-perovskite compounds.

### 2.4. Catalytic activity

The catalytic activity of the prepared perovskite samples was carried out in a continuous flow system comprising a quartz tubular reactor operating under atmospheric pressure on using 0.2 g of the prepared sample, which was diluted with 0.2 g of silicon carbide. A methanol/ethanol mixture (1 : 1) was fed into the reactor by a dosing pump with a flow rate of 2 ml per hour using nitrogen as carrier gas (45 cm^3^(NTP) per min). The reaction temperature was varied between 150 and 300 °C. The products such as acrolein, formaldehyde, acetaldehyde were analyzed using off-line flame ionization detection (FID) gas chromatography.

## 3. Results and discussion

### 3.1. Structural analysis

**Fig. 1:**
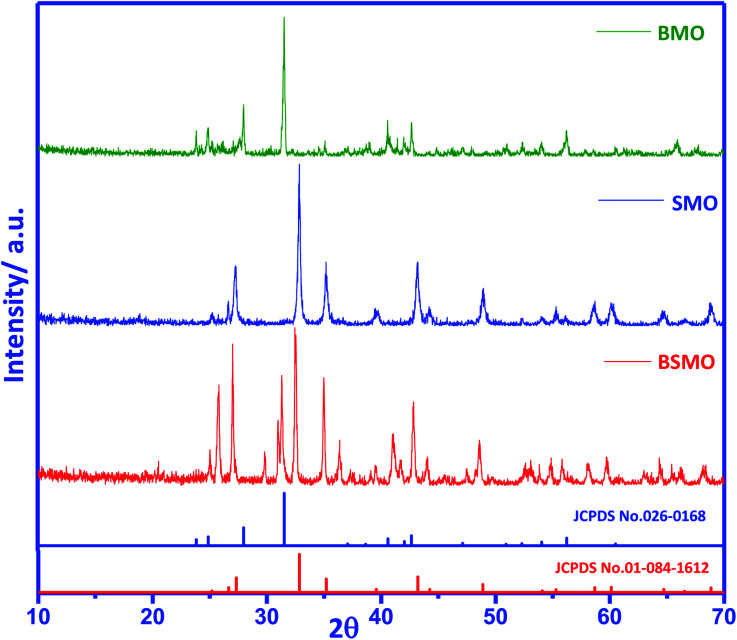
XRD patterns of BMO, SMO, and BSMO. Magnification of the XRD patterns to identify the shift of the main peaks.

The XRD pattern for the prepared samples BaMnO_3_, SrMnO_3,_ and Ba_0.5_Sr_0.5_MnO_3_ (Fig. 1) shows represent well-determine sharp peaks, which means that the prepared samples are crystalline in agreement with JCPDS standard data file no. 0260168,^18^ while SrMnO_3_ and Ba_0.5_Sr_0.5_MnO_3_ structure indexed to JCPDS standard data file no. 010841612.^19^ The diffraction patterns of Ba_0.5_Sr_0.5_MnO_3_ displays the light enlargement of the prominent peaks, and the main peak position 2*θ* (∼32.8°) progressively shift to lower 2*θ* values (∼32.4°), which confirmed the replacement of Sr^2+^ with Ba^2+^.^19^ The intensity peaks in the Ba_0.5_Sr_0.5_MnO_3_ sample illustrate mixed phases, *i.e.*, not totally in the hexagonal structure. Other distorted forms (orthorhombic, octahedral, tetragonal) may be possible in this case. Such structural variations are expected since the greater Ba^2+^ ion (1.47 Å) in the perovskite structure replaces the smaller Sr^2+^ ion (1.13 Å), which results in increased cell volumes and decreased crystallinity, which also confirms the substitution of ions.^20^ The crystal size quantified by Scherrer's equation is 25 nm for BaMnO_3_ and 39 nm for SrMnO_3,_ which decreases to 21 nm for Ba_0.5_Sr_0.5_MnO_3_ catalyst (Table 1).

**Table 1:** Surface area and XPS characterization data of the perovskite catalysts

Catalyst	*D*_xrd_ a	SSA^b^, m^2^ g^−1^	*V*_p_ b, cm^3^ g^−1^	Atomic concentration^c^ (%)	Binding energies^c^ (ev)			Active oxygen^c^ (%)	Mn^4+^/Mn^3+^	O_L_/Ba + Sr + Mn
				Ba : Sr : Mn	O_L_	O_ads_	O_hyd_			
BMO	25	15	0.03	40 : 00 : 56	528.81 (6265)	530.58 (6559)		51%	0.13	2.4
SMO	39	18	0.02	0 : 59 : 49	528.14 (8656)	530.05 (7357)	531.68 (2100)	40%	0.29	1.7
BSMO	21	20	0.14	43 : 24 : 47	528.63 (5857)	530.9 (6610)	532.63 (1270)	53%	0.34	1.6

a From Scherrer relationship.

b From BET theory.

c Surface elemental composition of the perovskite catalysts calculated from the peak area from XPS, N.B: areas under the peaks were exhibited between parenthesis.

Based on the tolerance factor (*t*) of BaMnO_3_, SrMnO_3_, and Ba_0.5_Sr_0.5_MnO_3_, which equals 1.097, 1.033, and 0.942, respectively, these structures can be explained as follows: if (*t*) is equal to one, the geometry of the composition is ideal cubic perovskite. Nevertheless, if (*t*) is minor larger than unity, as in BMO and SMO, hexagonal perovskite structures are formed. Cations in the A site are enlarged because there is an increasing amount of face-shared links between octahedrons of MnO_6_. The bond angle between Mn–O–Mn face-shared linkages is detected to be 90° in these structures. In case *t* was slightly decreasing from unity as in BSMO, as a consequence of twisting and sloping alterations of MnO_6_ octahedron, Mn–O–Mn bond angle decreases 180° to recompense small A-site cations.^21,22^

**Fig. 2:**
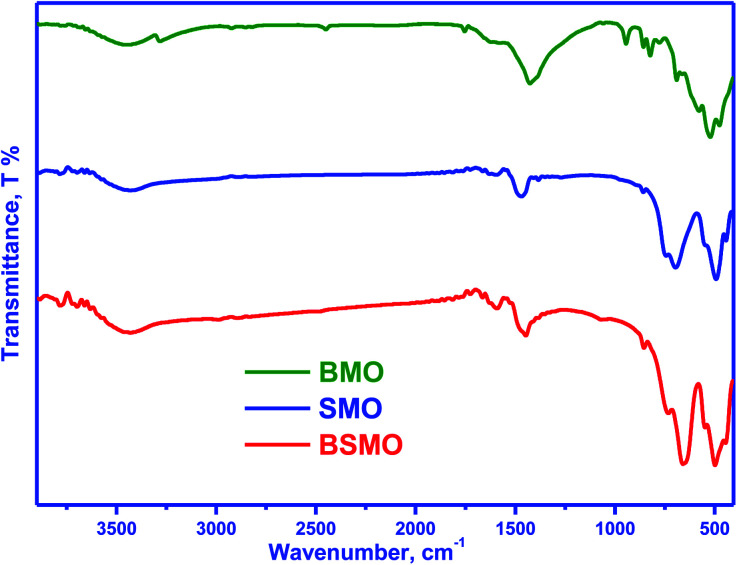
FT-IR spectra of BMO, SMO and BSMO.

Fig. 2 displays the infrared spectra of the synthesized perovskites. The peak seen in the wavenumber range 450–492 cm^−1^ corresponds to the bending mode, which is sensitive to change in the Mn–O–Mn bond angles. The peak assigned in the range 505–786 cm^−1^ is attributed to the stretching vibration of metal oxide (M–O) bonds in BMO. The peak at 1590 cm^−1^ can be related to the H–O–H bending vibration. The main absorption band around 600 cm^−1^ corresponds to the stretching of the metal–oxygen bond in the perovskite, which involves the internal motion of a change in Mn–O–Mn bond length in MnO_6_ octahedral, which certified the formation of metal oxide framework in the perovskites. The three peaks at 760, 696, and 510 cm^−1^ are ascribed to the stretching vibration of metal–oxide (M–O) bonds in SMO, matching well with the published literature. The peak centered at 1639–1649 cm^−1^ is due to the bending vibration of aerially adsorbed water. The weak broadband visible around 3447–3498 cm^−1^ indicates the stretching vibration of hydroxyl groups (OH or H_2_O) with an intramolecular hydrogen bond. The FTIR spectra for BSMO perovskite show a similar SMO pattern, with an increase in the intensity of the vibration frequency at 1628 cm^−1^ which is characteristic of Ba–O stretching mode, and the band at 850 cm^−1^ attributed to the stretching vibration of metal oxide (M–O) bonds in BSMO sample. Also, the broadening and increasing intensity of the peaks in the range of (400–600 cm^−1^) the position of the stretching vibration of metal oxide (M–O). That is confirming the doping of SMO by Ba ion.^23^

**Fig. 3:**
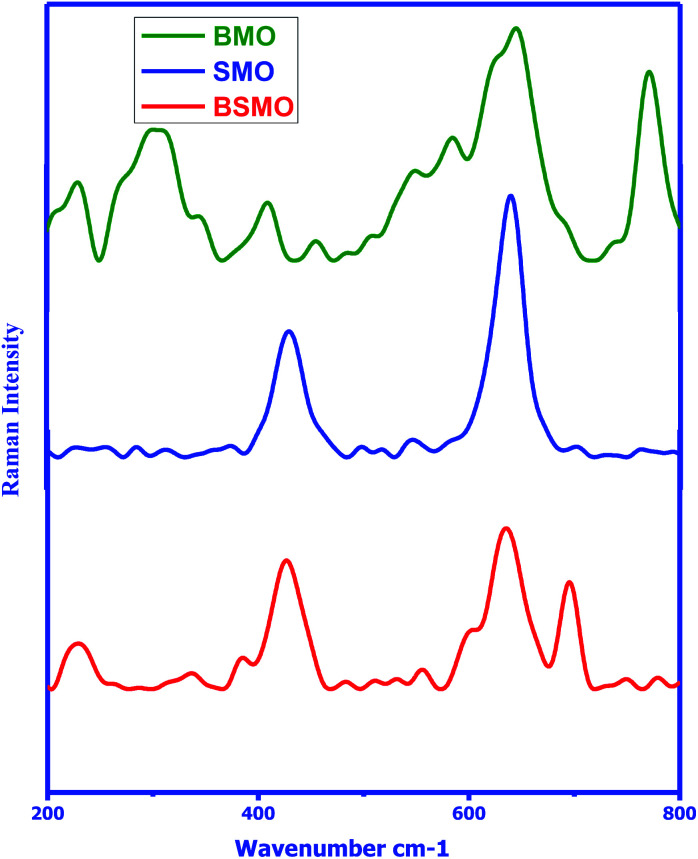
Raman spectra of BMO, SMO and BSMO.

The structure of the prepared samples was also confirmed *via* Raman spectroscopy in the wavenumber range of 200–800 cm^−1^ (Fig. 3) clarified the behavior of the Raman intensity in the spectral ranges of 370–445 cm^−1^ and 610–770 cm^−1^ which are characteristic for bonds of Mn–O–Mn. In BSMO, 695 cm^−1^ indicating Ba–O–Ba bonds. These data are matching well with previously reported papers.^24^ Thus, the FTIR and Raman investigations are agreed well with the results of XRD.

**Fig. 4:**
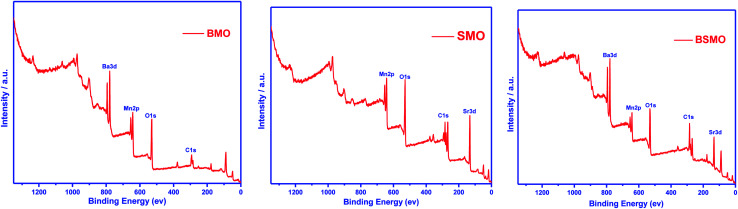
X-ray photoelectron spectroscopy (XPS) survey of BMO, SMO, and BSMO.

**Fig. 5:**
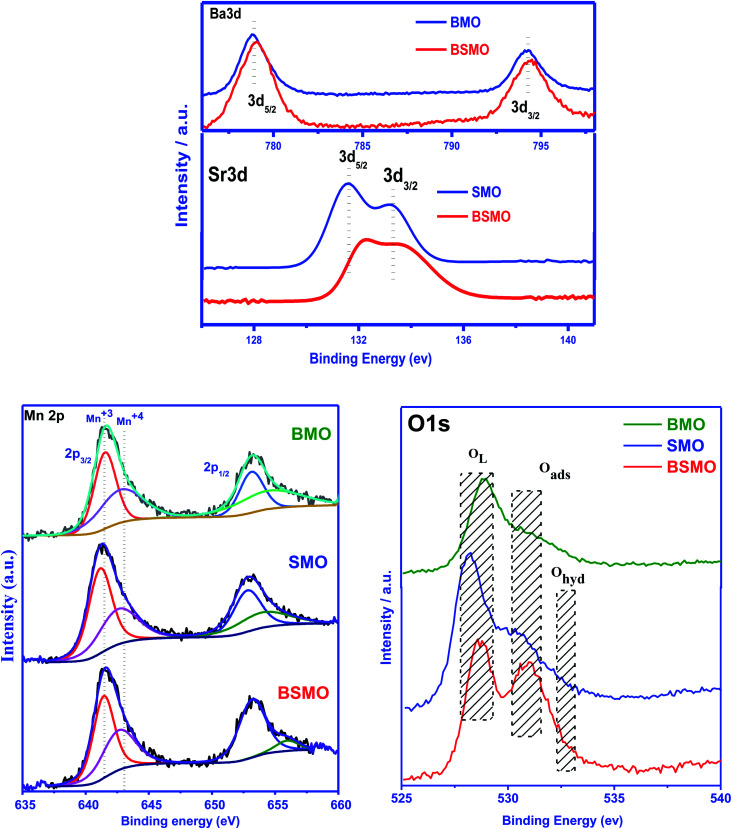
Ba 3d, Sr 3d, Mn 2p, and O 1s photoemission spectra of the BMO, SMO, and BSMO took in normal emission using non-monochromatic Al Kα X-ray source.

X-ray photoelectron spectroscopy (XPS) is a valuable tool to identify the composition and oxidation state of the ions in the perovskite structures. XPS patterns of BMO & SMO samples (Fig. 4 and 5) display the essentials two peaks. For BMO sample binding energies (B.E) at 778.7 & 794.2 eV attribute to Ba 3d_5/2_ due to Ba^2+^ in the perovskite lattice and Ba 3d_3/2_ related to the surface oxide/hydroxide, respectively. The two peaks at 131.61 & 133.38 eV for the SMO sample identified the binding energies for Sr 3d_5/2_ and Sr3d_3/2_. These values established the presence of Sr^2+^ species in the lattice and the SrO/Sr(OH)_2_ structure on the surface, respectively. This means that Ba and Sr in the samples display +2 valence on the catalyst surface.^25,26^ On the other hand, the binding energy position for the BMSO sample is slightly shifted may be related to the partial substitution of the lower electronegative element (Sr: 0.95) with a higher one (Ba: 0.89). At the same time, the Ba 3d lines are not affected, which emphasized that substitution occurs in SMO, not in BMO lattice, in good agreements with XRD data.

From the literature, the assignment of the manganese oxidation states by XPS is a difficult task.^27^ In Fig. 5, the peak maximum of the Mn 2p_3/2_ transition suggests the occurrence of Mn^3+^ and Mn^4+^ in all the prepared samples. The Mn 2p_3/2_ band has been deconvoluted, and peaks at around 641.3 eV and 643.1 eV are ascribed to Mn^3+^ and Mn^4+^, respectively. It affirms the presence of +3 and +4 valence states of Mn on the surface of all samples. From the area of the corresponding peaks, the ratio Mn^4+^/Mn^3+^ was calculated and be lower than 1 for all the samples indicate that Mn^3+^ is the main oxidation state, the Mn^4+^/Mn^3+^ ratio for BMSO is the lowest value, which refers to the substitution of Ba with Sr promotes an increase of Mn^3+^ amount. Based on the BaMnO_3_ & SrMnO_3_ stoichiometric formula, Mn^4+^ has to be the predominant manganese oxidation state in the samples. Consequently, an increase of the Mn^4+^ amount would be expected to compensate for the additional defect of the positive charge (due to Sr substitution). However, XPS results reveal the opposite effect as the Mn^3+^ amount increase. Thus, electroneutrality must be achieved by generating oxygen vacancies by forming a non-stoichiometric (oxygen-deficient) perovskite ABO_3−*α*_. Furthermore, the highest amount of oxygen vacancies must be generated in the BSMO sample as it signified the lowest Mn^4+^/Mn^3+^ ratio.

Fig. 5 depicts the O 1s spectra of the prepared samples BMO, SMO, and BSMO. The O 1s spectra (SMO sample) with its signals at 528.1, 530.1, and 532.26 eV are ascribed to lattice oxygen (O^2−^), surface adsorbed oxygen (O_2_, O^−^), and surface carbonates and/or hydroxyl groups (O_hyd_), respectively.^28^ The release from oxygen lattice (O^2−^) is usually caused by the redox binding of metal ions on the catalyst's surface. Indeed, the catalyst oxygen vacancy density sites are attributed to the release of adsorbed oxygen (O_2_, O^−^) species.^29^ We can also be observed that the release of oxygen species is attributable to variations in electron negativity between metal and oxygen which means that the metal and oxygen interactions are bindings.^30^ For alcohol partial oxidation reactions to corresponding aldehydes, higher content of (O_2_, O^−^) species is needed for that low-temperature reactions. As shown in Table 1, the percentages quantity of active oxygen of the samples, BMO, SMO, and BSMO, are found to be 51%, 40%, and 53%, respectively. It is clarified that the calculated amount of active oxygen species was higher for doped samples than the undoped ones. La_1−*x*_K_*x*_Co_1−*y*_Cu_*y*_O_3_ catalysts with A and B cations substitution were tested, resulting in a rise in the surface oxygen groups correlated with oxygen vacancy generation.^31^

Furthermore, Zhan and the team found that when lanthanum substitutes for LaMn_0.5_Cu_0.5_O_3_ perovskite, an increase in the O_ads_/O_L_ ratio suggests a greater amount of oxygen defects catalysts.^32^ Similarly, the lower amount value of the O_L_/(Ba + Sr + Mn) ratio increases the oxygen vacancies in prepared perovskites. In addition, BSMO catalyst is the perovskite with the highest number of structural defects, as is expected based on the analysis of the Mn 2p_3/2_ spectra. Thus, the sample BSMO might be an excellent catalytic activity because of its excellent redox properties and higher surface-chemisorbed oxygen species.

**Fig. 6:**
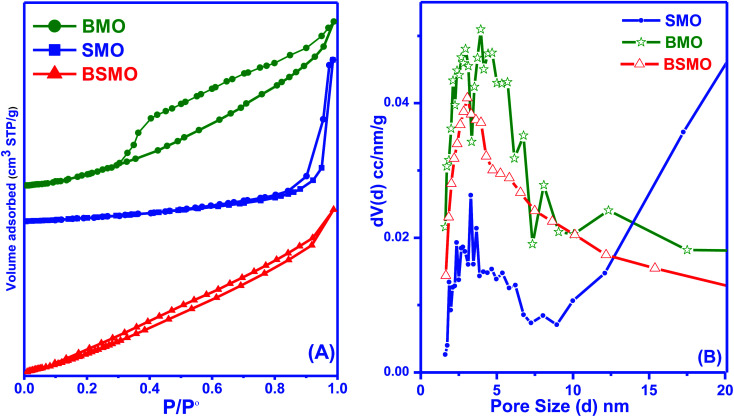
BET surface area (A) and pore size distribution analysis (B) of BMO, SMO, and BSMO catalysts.

The textural parameters of the prepared nanostructured materials are listed in (Table 1 and Fig. 6) represented that all samples exhibited that a classical type IV isotherm, which was the characteristic of the mesoporous structure. The presence of H2 type hysteresis loops in the isotherms suggested the appearance of ink-bottle pores with narrow entrances and large cavities. The mean diameter and total volume of the nanostructures (Table 1) in the range of 1–10 nm by using the BJH analysis indicating the porous mesoporous structure.

**Fig. 7:**
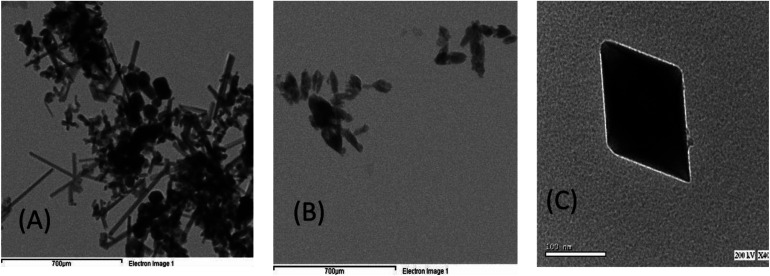
TEM images of hierarchically nanostructured BMO (A), SMO (B), and BSMO (C).

The morphology and microstructure of the sample were more explored by TEM (Fig. 7). Fig. 7A and B represent TEM images of the hierarchically nanostructured (BMO and SMO) perovskite samples, in which one can observe an individual nanorod with a smooth surface. In contrast, Fig. 7C (BSMO) shows the edges of the nanosheets built up by nanorods. That is similar to,^33^ who observed the same behavior on preparing hierarchically porous LaFeO_3_*via* the hydrothermal method.

The number, strength, and strength distribution of acid/base sites were analyzed by TPD of NH_3_ and CO_2_ probe molecules, respectively. It is important to note that ammonia is suitable to quantify Brønsted acid sites (transfer of a proton from surface hydroxyls) and Lewis sites (coordination with an electron-deficient atom such as a metal cation).

**Fig. 8:**
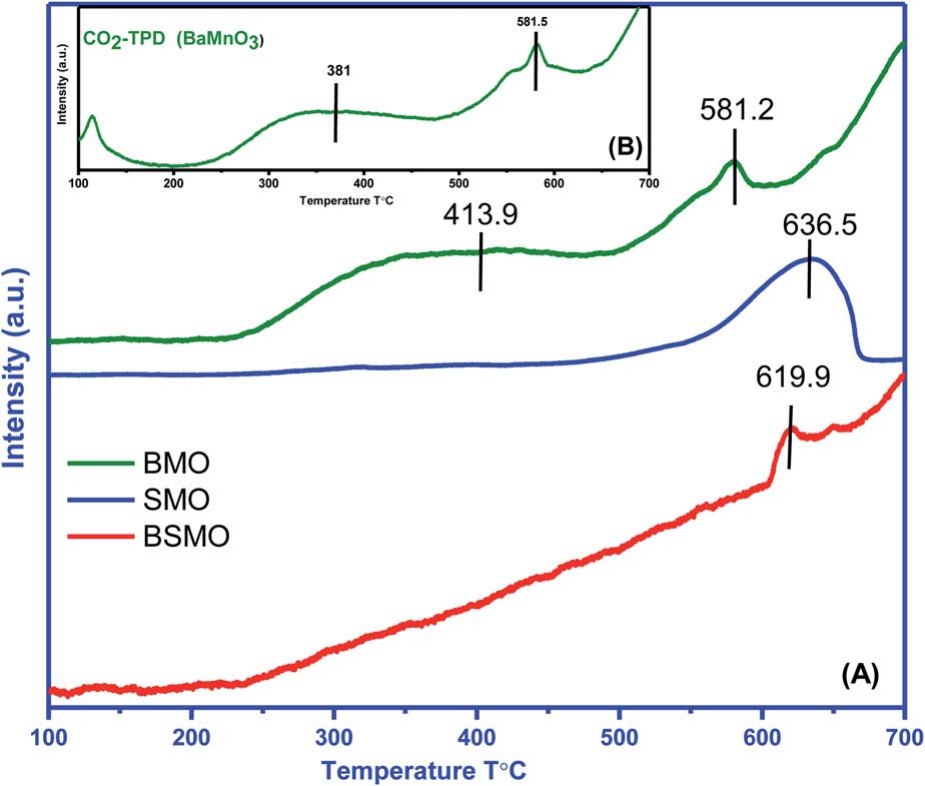
NH_3_-TPD (A) of BMO, SMO, and BSMO catalysts, inset the CO_2_-TPD (B) of BMO.

**Fig. 9:**
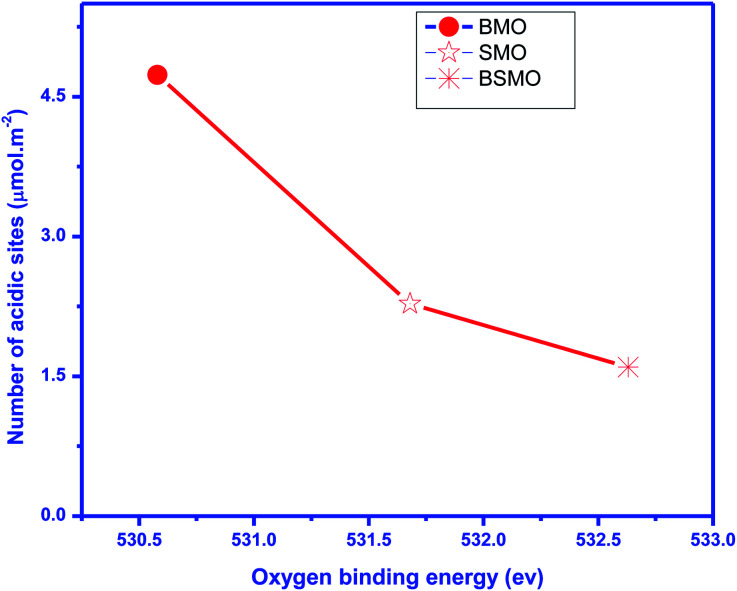
Oxygen binding energy obtained by XPS *vs.* a number of total surface acidic sites (expressed in μmol m^−2^).

According to previous reports,^34^ the NH_3_ molecules coordinated to the Lewis acid sites exhibit higher thermal stability than the NH_4_^+^ ions bound to the Brønsted acid sites; herein, the mid-strong acid sites could mainly be the Lewis acid sites. For BMO, two desorption peaks were detected and attributed to the mid-strong acid sites (200–500 °C). Similarly, the number of CO_2_ desorption peaks (Fig. 8) was observed to be the same as the number of NH_3_ desorption peaks on BaMnO_3_. The total amount of acidity and basicity are 71 and 79 μmol g^−1^, respectively. So, catalysts displayed both acidic and basic characters. For the SMO and BSMO, the ammonia desorption peaks position 636.5 and 619.9, and the total acidity is 41 and 32 μmol g^−1^, respectively. By comparing the amount of NH_3_ desorption, it was concluded that the introduction of Ba played a positive effect on providing mid-strong acid sites. It is worth noting that BSMO has the least amount of strong acidic sites of all the prepared samples. Indeed, as displayed in Fig. 9, the O 1s binding energy (from XPS) decreases with an increasing number of acidic sites per surface area square meters. However, lower O 1s binding energy indicates an electron-rich element, which means more basic sites agree with Védrine *et al.*,^35^ proposal. All these results approve the amphoteric character of all samples.

### 3.2. Catalytic activity

**Fig. 10:**
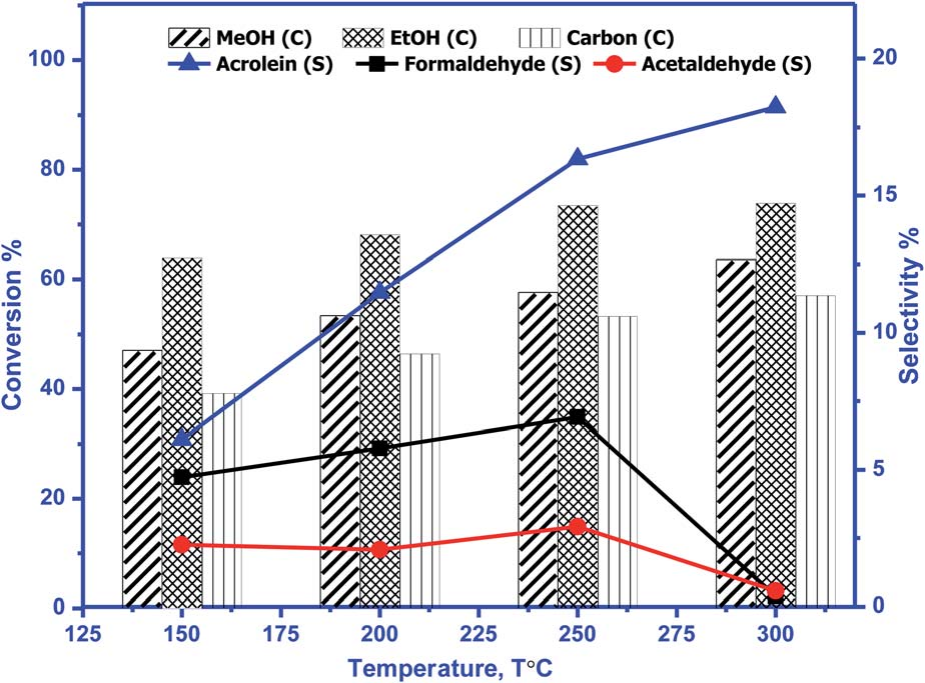
Conversion (*C*) of methanol, ethanol, and carbon & selectivity (*S*) of formaldehyde, acetaldehyde, and acrolein and product distribution for BaMnO_3_ (BMO). The experimental conditions: temperatures (150–300 °C) & LHSV (10 h^−1^) methanol/ethanol mixture = 1 with flow rate of 2 ml per hour and N_2_ flow rate 45 ml min^−1^.

**Fig. 11:**
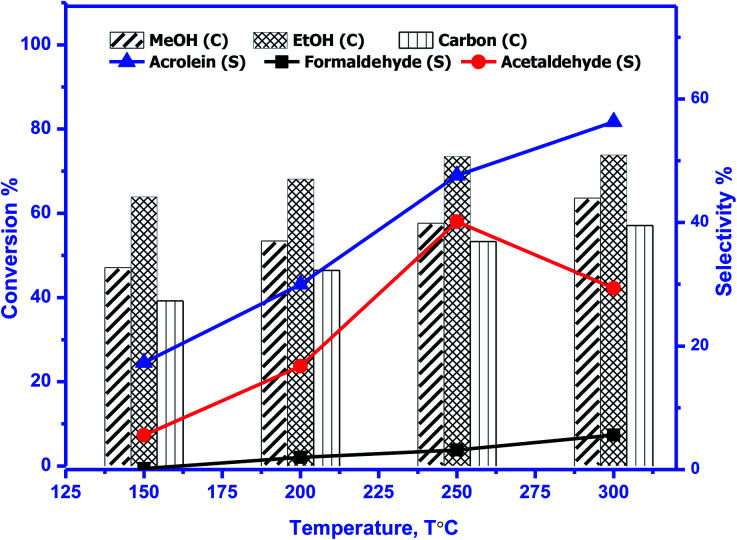
Conversion (*C*) of methanol, ethanol, and carbon & selectivity (*S*) of formaldehyde, acetaldehyde, and acrolein and product distribution for SrMnO_3_ (SMO). The experimental conditions: temperatures (150–300 °C) & LHSV (10 h^−1^) methanol/ethanol mixture = 1 with flow rate of 2 ml per hour and N_2_ flow rate 45 ml min^−1^.

**Fig. 12:**
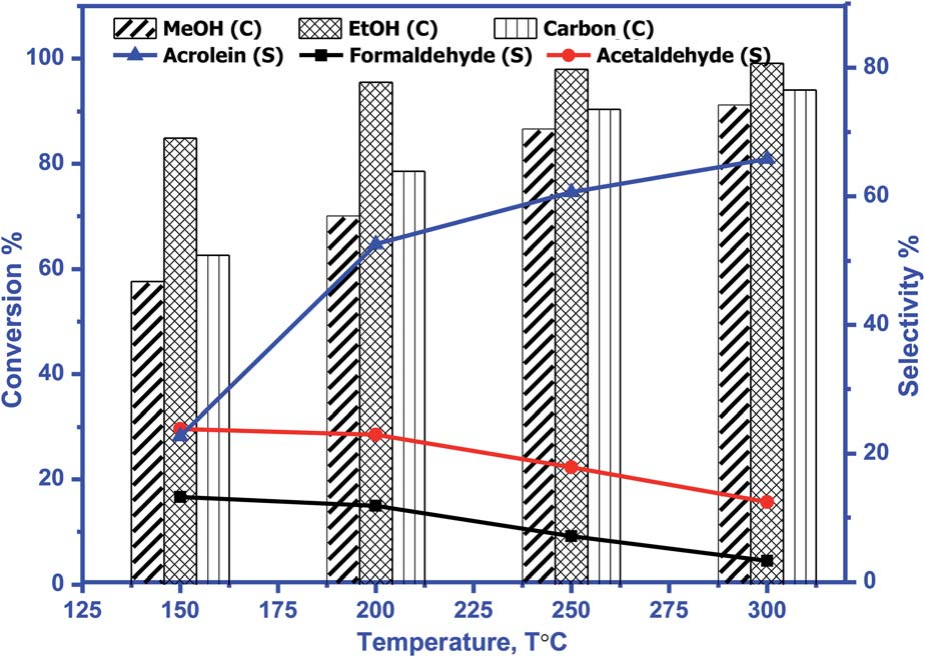
Conversion (*C*) of methanol, ethanol, and carbon & selectivity (*S*) of formaldehyde, acetaldehyde, and acrolein and product distribution for BaSrMnO_3_ (BSMO). The experimental conditions: temperatures (150–300 °C) & LHSV (10 h^−1^) methanol/ethanol mixture = 1 with flow rate of 2 ml per hour and N_2_ flow rate 45 ml min^−1^.

The catalytic activity of prepared perovskite samples was evaluated using a mixture of bio-alcohols methanol and ethanol in the presence of carrier (N_2_) with conditions LHSV = 10 h^−1^, temperature range = 150–300 °C, MeOH/EtOH = 1 molar ratio. The effect of temperature on mixed alcohol conversion, product distribution, and selectivity for prepared samples is graphically illustrated in Fig. 10–12.

In our study, reaction conditions, a wide range of different reaction products of methanol and ethanol conversion can be classified as follows: (a) C–C coupling (*e.g.*, C_3+_ alcohols; C_3+_ aldehydes; C_3+_ esters); (b) non-C–C coupling (*e.g.*, formaldehyde, acetaldehyde, ethyl acetate). From the data in the figures, we can see that an increase in temperature from 150 to 300 °C affects pointedly on methanol and ethanol conversions. Methanol conversion was reached 64, 63.2, 90.2%, while ethanol conversion was reached 74, 73.4, 98.4% using BMO, SMO, and BSMO samples, respectively. As clarified, a good correlation between the conversion of alcohols and metal composition on the perovskite surfaces. The most essential obtained products are acrolein, acetaldehyde, and formaldehyde. In addition to traces of other tiny products. The low carbon balance (in the 57–93%) results from coke deposition on the catalyst surface.^1^ The absence of crotonaldehyde, acetaldehyde *via* self-aldolization (2CH_3_–CHO → CH_3_–CH–CHO), may be explained as follows: from thermodynamic view at a temperature range between 150 and 400. Acrolein is the more favored product formed than crotonaldehyde. Due to alcohol oxidation to aldehydes, the partial pressure of water vapor is even higher, inhibiting the self-aldolization reaction, and a pronounced product acrolein was formed.^1^ In other words, in the absence of oxygen, as in our study, both acetaldehyde and formaldehyde were adsorbed (creating two carbocations) and further reacted to form acrolein.

There is a positive correlation between catalytic activity and Mn^4+^/Mn^3+^ ratio on the surface (obtained from XPS). Mn^4+^ acts as stronger Lewis acids than Mn^3+^ and weak Lewis acid sites are required for this reaction. Therefore, BSMO with a low Mn^4+^/Mn^3+^ ratio exhibits high activity while BMO low catalytic activity. The loss of an electron (Mn^3+^) results in a significant migration of the unstable interstitial oxygen, which escapes from the lattice and transforms into active oxygen (O^−^). Similarly, doping of Sr^2+^ in a LaMnO_3_ perovskite has been described to present a mixture Mn^3+^ and Mn^4+^ states, where Mn^3+^–O bonds have different ionic strength than Mn^4+^–O bonds and consequently formed oxygen vacancies.^36^ Recent studies demonstrate that basic sites are required for catalyzing cross-condensation because they can activate acetaldehyde by abstracting a hydrogen atom from the α-position of carbonyl.^6^ In addition, weakly acidic sites can activate formaldehyde by increasing the C atom's electronegativity.^9^ Another report used molybdenum dopped copper ferrite for oxidative coupling of primary alcohols, demonstrate that the aldehyde formation by dehydrogenation or by oxidation needs the presence of moderately basic sites (M^2+^–O^2−^).^37^

For selectivity, acidic sites exhibit a strong influence on selectivity; the cross-selectivity seems to be controlled by acidity rather than basicity. On the other hand, using amphoteric catalysts as in our work, acrolein production is improved, and carbon oxides production is reduced, consequently allowing the catalyst to work at higher temperatures.^8^

As mentioned in TPD analysis, the prepared perovskites have acidic and basic sites and are considered amphoteric catalysts. BSMO exhibits a high basic character which explains its high catalytic activity rather than BMO and SMO. BSMO displays moderate acidic and basicity character among the others, which elucidate high acrolein selectivity at relatively low temperature (200 and 250 °C), which reached ∼52 and 60%, respectively.

The formation of acrolein *via* cross condensation between formaldehyde and acetaldehyde can be summarized *via* the following equations (eqn (5)–(8)):

5 O^−^(Surf) + O=CHCH_3_ → (Surf)–O–CHO^−^–CH_3_

6 (Surf)–O–CHO^−^–CH_3_ + O=CH_2_ → (Surf)–O–OHC–CH_2_O^−^–CH_3_

7 (Surf)–O–OHC–CH_2_O^−^–CH_3_ ↔ (Surf)–O–O^−^–C–CH_2_OH–CH_3_

8
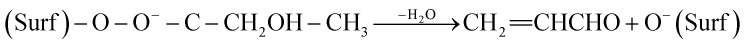


The methyl ethyl ether selectivity is higher at low temperatures. The catalyst order BMO > BSMO > SMO decreases with increasing temperature, in which dehydration reactions occur faster than other reactions. One alcohol molecule is adsorbed on the weak Lewis acid sites *via* the oxygen of the OH group. The second alcohol molecule is adsorbed on the strong Lewis basic site *via* the H of the OH group, resulting in forming an alkoxide on the surface, which reacts to form corresponding ether.^38^ On the contrary, methyl acetate formation increases with increasing temperature. Indeed, the BSMO has a higher selectivity for methyl acetate, reaching 18% at 300 °C. The selectivity is explained by the formed acetaldehyde with the newly formed methanol molecule *via* oxidative esterification (non-C–C coupling) to form methyl acetate.

For all samples, methyl acetate reached the plateau or slowly increased in selectivity with increasing temperature due to complete conversion of methanol as in BSMO or methanol decomposition favored at high temperatures. The increase in the selectivity of propanol and butanol alcohols is consistent with their involvement in esterification reactions, as evidenced by the concurrent increase in the selectivity of ester. Moreover, the presence of aldehyde accelerates the ester formation and, at the same time, prevents the oxidation of the secondary aldehyde to acid.

**Fig. 13:**
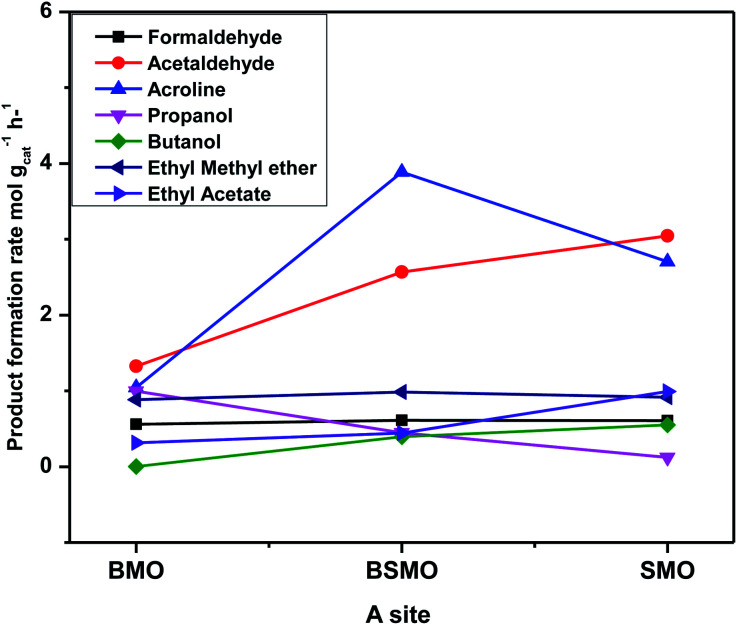
Product formation rate *vs.* all prepared catalysts with different A. The experimental conditions: temperatures (250 °C) & LHSV (10 h^−1^) methanol/ethanol mixture = 1 with flow rate of 2 ml per hour and N_2_ flow rate 45 ml min^−1^.

Fig. 13 illustrates that the doping of SMO with Ba^2+^ in BSMO attained the highest rate of C–C coupling products relative to non-C–C coupling at 250 °C. In conclusion, the observed results suggested that the prepared BMO, SMO, and BSMO samples are more favorable for C–C coupling products with low production of non-C–C coupling and negligible CO_*x*_ byproducts.

## 4. Characterization of used catalysts

**Fig. 14:**
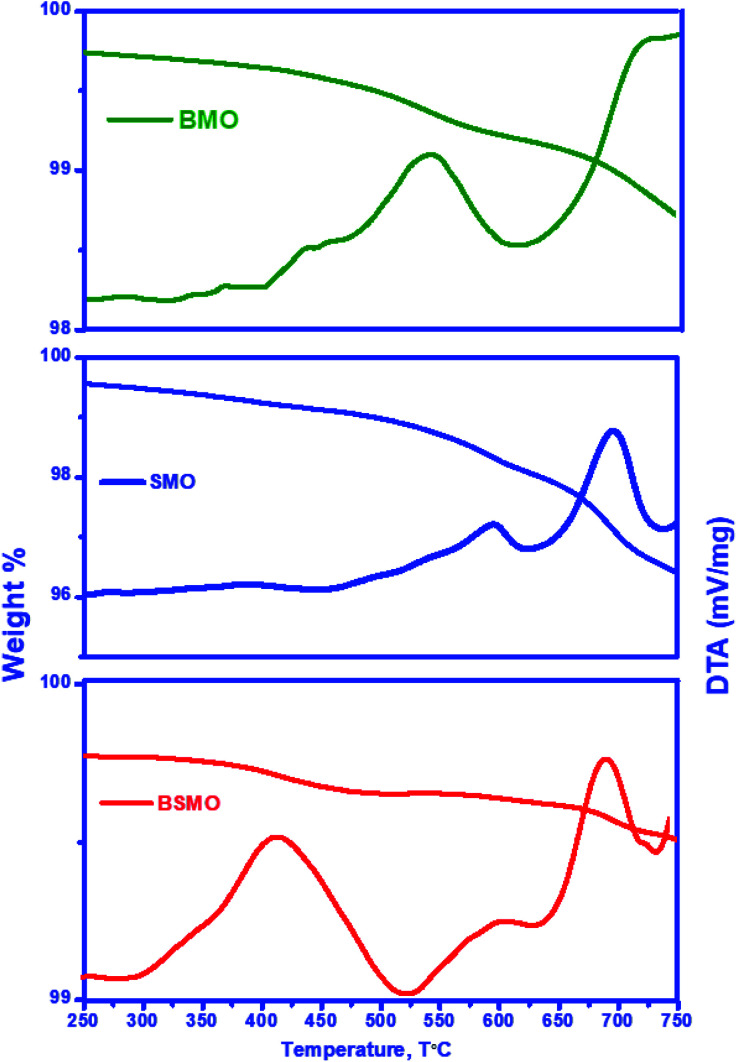
TGA and their derivative spectra of used BMO, SMO, and BSMO catalysts after oxidative coupling reaction of methanol and ethanol.

The types of carbon species formed on the used BMO, SMO, and BSMO catalysts were examined using a thermogravimetric analyzer (TGA). Their TGA profiles and derivatives are shown in (Fig. 14). The initial loss below 300 °C in all catalysts is due to removing moisture, and CO_2_ adsorbed, followed by the loss of easily oxidized carbonaceous species.^39^ The used BSMO catalysts profile exhibits an exothermic peak at around 415 °C, attributed to physisorbed carbonaceous products (softcore). In comparison, BMO and SMO profile exhibits the peaks at higher temperature 540 and 590 °C respectively. This associated with filamentous hard coke followed a major decrease in the sample's weight (∼1.5 and 2%), showed the gasification of the bulky carbonaceous species or hard coke, generating CO and CO_2_ (CO_*x*_).^40^ In addition, the peak at the high-temperature range of 600–700 °C is attributing to graphitic carbon species. This graphitic carbon is a non-reactive coke that infrequently reacts with oxygen.^41^

**Fig. 15:**
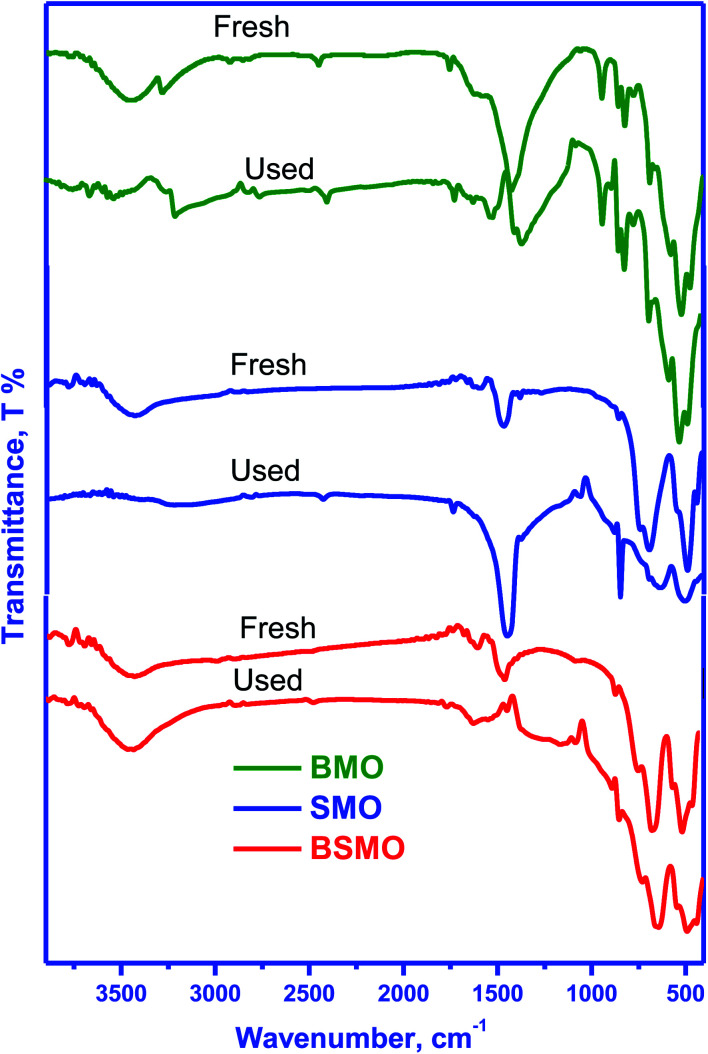
FT-IR spectra of fresh and used BMO, SMO, and BSMO catalysts after oxidative coupling reaction of methanol and ethanol.

Fig. 15 shows the FT-IR spectra of fresh and used after oxidative coupling reaction of methanol and ethanol. There is no noticeable difference in the spectra for the fresh and used catalysts. The intensities of all bands decreased, probably due to coke deposition on the perovskite surface. This behavior is not found to a great extent in the case of a BSMO catalyst. The coke formation could further verify this, as shown in the TG curves.

**Fig. 16:**
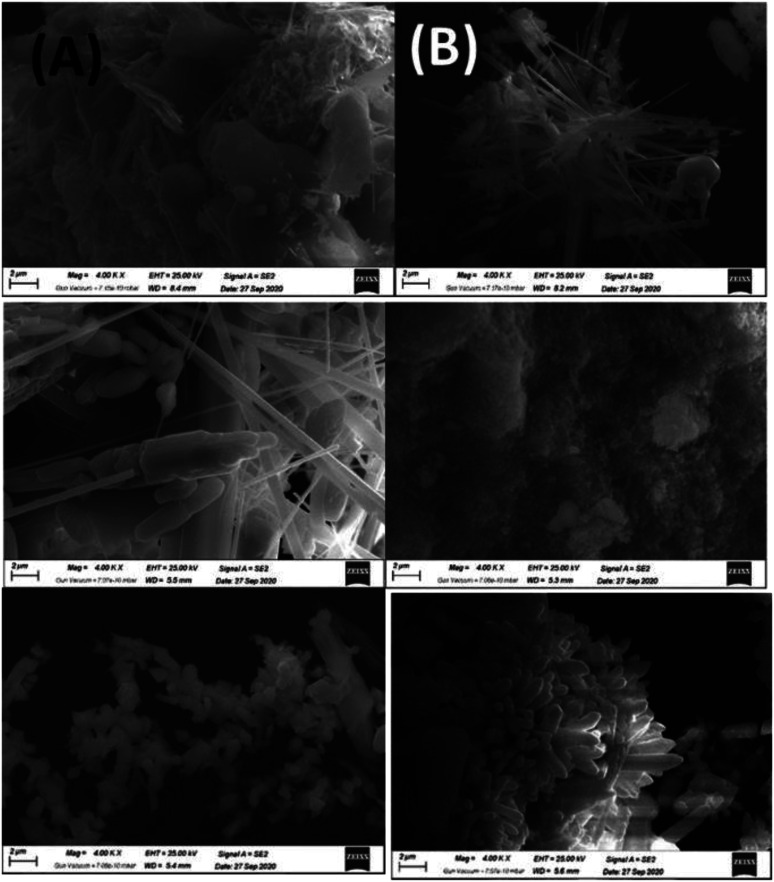
FESEM images of BMO, SMO and BSMO perovskite catalysts fresh (A) and after (B) oxidative coupling of mixed alcohol reaction.

SEM images obtained at 4000 magnification are shown in (Fig. 16A), revealing the effect of the change in A element in ABO_3_ perovskite on uniformity, homogeneity, and surface morphology of the perovskite samples. All samples show good homogeneity having almost uniform shapes. The grain boundaries are sharp, with most of the grains have nanorods with a diameter in the nanometer range and a length of 2 μm. The average grain diameter for the BSMO is the least one of the fresh catalysts showing that grain size is highly doping dependent. Most grain's plane and sharp faces also show the crystallographic texture and well-oriented growth samples.^22^ The images also show the walls of macropores, which were assembled by a large number of nanoparticles with the formation of nanovoids, and it was apparent confirm that perovskite samples possessed hierarchically pore structure; this would make reactants more diffuse and increase the contact effectiveness between reactants and catalysts so that improved catalytic performance could be achieved.^33^

All surfaces have been rearranged to take on more regular forms after the reaction as shown in (Fig. 16B). In contrast, SMO has modified all surface morphologies to reflect further particle deformation and agglomeration, mainly because of the problematic reaction conditions during the process, so that it generated no aggregates of regular shapes or structures.

**Fig. 17:**
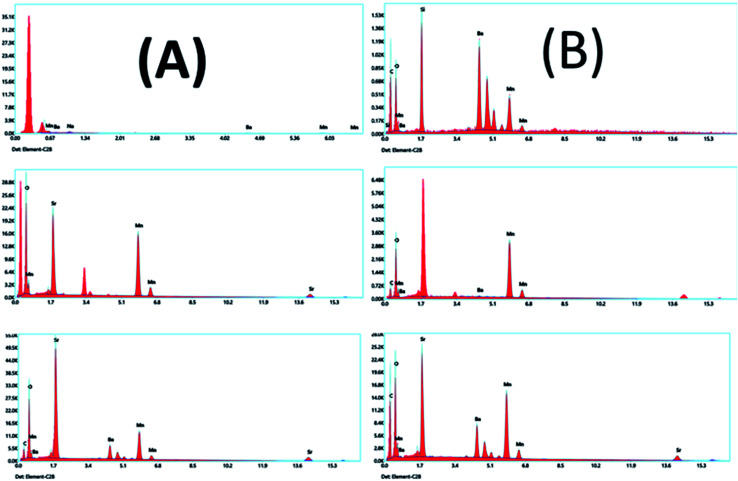
EDX analyses of BMO, SMO, and BSMO perovskite catalysts fresh (A) and after (B) oxidative coupling of mixed alcohol reaction.

EDX is a technique used to identify the elemental composition of the typical product. The EDX spectra established in all the samples have no impurity element present in the samples (Fig. 17A and B). The elements present in the respective spectra are correct stoichiometric ratios, precisely following empirical formulas of the corresponding composition. The EDX results prove that only three types of elements, Ba or Sr, Mn, and O, in the BMO and SMO samples. And four elements Ba, Sr, Mn, and O in the BSMO sample. There are some minor peaks related to C (in fresh catalyst) because of the sticking tape used to hold the samples in place. It related the carbon peaks that appear in the EDX spectra to coke deposition,^43^ as confirmed in TGA and FTIR analysis for used catalysts.

Table 2 shows the comparison of different catalysts towards acrolein selectivity *via* oxidative coupling of bio-alcohols mixture.

**Table 2:** Comparison of different catalysts towards acrolein selectivity *via* oxidative coupling of methanol and ethanol mixture

Catalyst	Reaction temperature, °C	Acrolein selectivity %	Reference
Commercial FeMoO_*x*_	300	44	42
FeMoLa_2.0_	320	42	44
Calcined hydrotalcites samples with various Mg/Al molar ratios	305	33	4
12A_l2_O_3_·1MgO	305	24	43
Hierarchically porous perovskite	300	60	This work

## 5. Conclusion

The physico-chemical properties of hierarchically porous perovskite catalysts were determined by XRD, FTIR, Raman spectroscopy, XPS, N_2_ sorption, TEM, SEM, TGA, and NH_3_–CO_2_-TPD. Also, the role of A (Ba or/and Sr) position variation in AMnO_3_ perovskite catalysts has been studied in the oxidative coupling of primary alcohols performed at atmospheric pressure using a methanol/ethanol equal 1. These characteristics were associated with catalytic activities for the aldolization reaction (to form acrolein), promoted by coordination between the acids and basic sites. The balance of acidic and basic properties of the catalysts is critical in the production of acrolein. Of the catalysts studied, BaSrMnO_3_ demonstrated the highest performance, followed by SrMnO_3_ and BaMnO_3_. The high activity was attributed to the significantly faster formation of C–C bonds in BaSrMnO_3_ than in other materials. Acrolein, acetaldehyde, methanol, ethanol were the main products found with each catalytic test. Crotonaldehyde was not formed during the reaction, indicating more resistance to self-condensation kinetics than the cross-condensation reaction under current reaction conditions. In general conclusion, these catalysts propose promising yields of acrolein, making it a superb choice to be effective in a viable process.

## Conflicts of interest

There are no conflicts to declare.

## Supplementary Material
